# Transparent Wood-Based Materials: Current State-of-the-Art and Future Perspectives

**DOI:** 10.3390/ma15249069

**Published:** 2022-12-19

**Authors:** Alberto Mariani, Giulio Malucelli

**Affiliations:** 1 Department of Chemical, Physical, Mathematical and Natural Sciences, University of Sassari, Via Vienna 2, 07100 Sassari, Italy; 2 Department of Applied Science and Technology, Politecnico di Torino, Viale Teresa Michel 5, 15121 Alessandria, Italy

**Keywords:** transparent wood, lignin, transmittance, refractive index, haze, aesthetic wood, smart glazing, energy-saving applications

## Abstract

Human history is largely characterized by the massive use of wood, the most well-known natural composite material, possessing unique thermal, mechanical, and environmental features that make it suitable for several applications, ranging from civil engineering, art, and household uses, to business uses (including furniture, stationery, shipbuilding, and fuel). Further, as a renewable and recyclable biomass, wood perfectly matches the current circular economy concept. However, because of its structure and composition, wood is not transparent: therefore, the possibility of removing the embedded lignin, hence limiting the light-scattering phenomena, has been investigated over the last ten to fifteen years, hence obtaining the so-called “transparent wood (TW)”. This latter represents an up-to-date key material, as it can be utilized as obtained or further functionalized, combining the transparency with other features (such as flame retardance, energy storage ability, and environmental protection, among others), which widen the potential (and practical) applications of wood. The present manuscript aims at summarizing first the current methods employed for obtaining transparent wood, and then the latest achievements concerning the properties of transparent wood, providing the reader with some perspectives about its novel functionalizations and applications.

## 1. Introduction

Among the current interests of modern society, the seeking of bio-sourced renewable and recyclable materials, able to replace their fossil-based counterparts partially or totally in different application sectors, is becoming very important and impacting materials science and technology. In this context, wood entirely fulfils these requirements, notwithstanding its important role as a carbon sequestrator [1].

As clearly reported in the literature, the chemistry of wood is mainly based on the combination of a few elements (namely carbon, hydrogen, and oxygen), which are organized in three main structures (i.e., cellulose, hemicellulose, and lignin), hence giving rise to the formation of a bio-composite material, possessing high mechanical strength and toughness, low density, and ease of processing [2,3]. Undoubtedly, structure and composition mainly affect the overall properties of wood, notwithstanding that the specific mechanical features are determined by the crystallization propensity of cellulose, and in particular the so-called crystalline region of wood, in which the linear macromolecular chains of cellulose are highly arranged in parallel with each other, in contrast to the amorphous region, characterized by high cellulose disorder [4]. Further, wood is a multifaceted material, characterized by complex hierarchical structures, high anisotropy due to the presence of open channels aligned in the growth direction, peculiar micro-, meso-, and macro-porosity, and opacity [5,6].

Despite its exploitation over a wide application range, wood suffers from several issues that somewhat limit its potential uses. In particular, the high anisotropy and the presence of OH groups account for high water sorption and subsequent expansion, hence favoring the formation of cracks and the occurrence of shrinkage phenomena, leading to the overall worsening of its quality and performance [7]. To limit these drawbacks, several approaches have been exploited so far, involving both chemical and physical treatments. As an example, Rousset and co-workers demonstrated the effectiveness of short-term pyrolysis treatments carried out under mild conditions (i.e., inert gas environment and temperatures between 160 and 250 °C) for enhancing the dimensional stability and durability of wood, thanks to the occurrence of hemicellulose degradation phenomena and the partial removal of both free and bound water [8]. Another proposed approach refers to high-intensity microwave treatments performed on woods possessing high moisture content: the high energy transferred by the microwave source gives rise to very rapid moisture evaporation that in turn increases the pressure inside the wood cell cavities, hence making the “swollen” biocomposite suitable for further physico-chemical modifications [9].

Further, several chemical treatments have been proposed so far for wood: they usually involve selected chemical products able to either interact (i.e., react) with lignin, hemicellulose, and cellulose or to fill the semi-closed wood cells, thus providing new thermal, mechanical, and optical features. As a result, the chemical-modified wood usually shows enhanced properties, specifically referring to its flame retardance, dimensional stability, hardness, electrical behavior, mechanical strength, and abrasion resistance, among others [10,11,12,13]. In this context, both as a new material (to investigate as it is, also after densification [14]) and a starting intermediate product for further modifications/functionalizations, transparent wood, from its discovery and morphological characterization in 1992 by S. Fink [15], started to acquire great importance as a new material with high potentialities. However, this new concept was somewhat abandoned until 2016, when two research groups, one from the KTH Royal Institute of Technology (Sweden) [16] and the other from the University of Maryland (USA) [17], concurrently rediscovered the potential of transparent wood. Since then, the delignification methods have been considerably improved, as well as the physico-chemical approaches for its further functionalization. As a result, nowadays, transparent wood can be considered a new, sustainable, smart “building block” for the design of novel functional and structural systems, showing interesting and impactful uses in different advanced sectors, ranging from transparent solar cell windows and optoelectronic components to light-transmitting buildings and structural materials for the automotive industry. This review is aimed at providing the reader with an up-to-date overview of the recent progress in transparent wood-based materials, highlighting their current potential in different advanced sectors, the latest achievements, and, finally, giving some perspectives about further developments for the forthcoming years.

## 2. Making Objects Transparent

**Figure 1:**
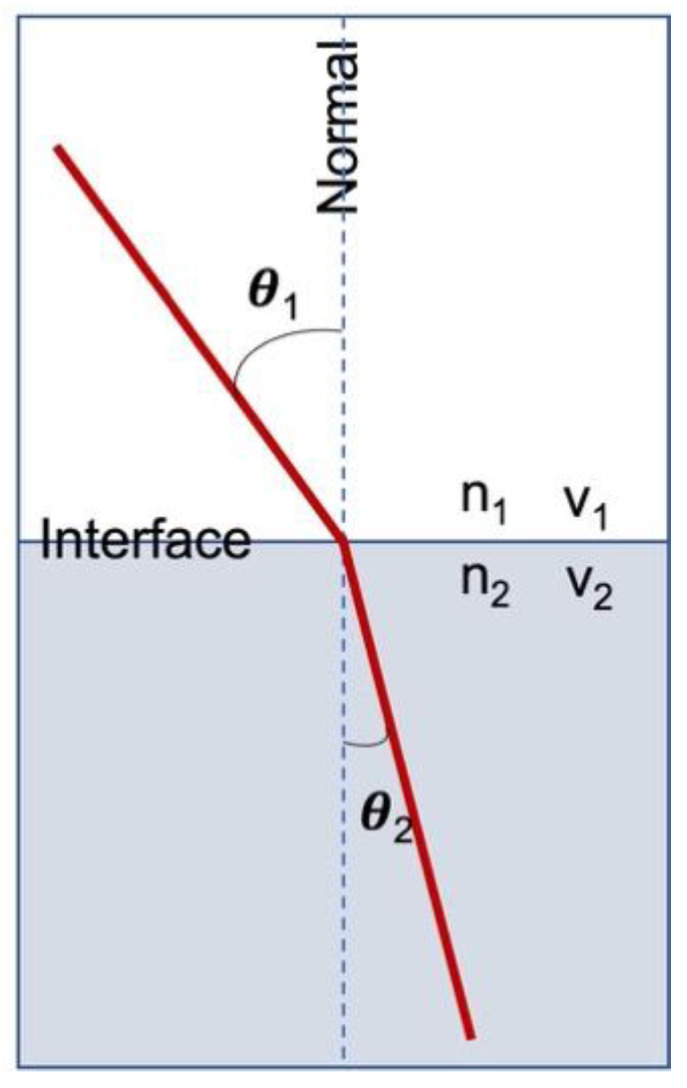
Refraction of light at the interface between two media of different refractive indices, with n_2_ > n_1_. Since the velocity is lower in the second medium (v_2_ < v_1_), the angle of refraction θ_2_ is less than the angle of incidence θ_1_.

Transparency is the physical property of allowing light to pass through a material without appreciable light scattering. On a macroscopic scale, in which object dimensions are much larger than the wavelength of light, photons can be said to follow Snell’s law (Figure 1).

On the other hand, translucent objects also allow light to pass through, but not necessarily by following Snell’s law. If a translucent material is composed of components with different refractive indices (RIs), photons can be scattered over one of the two interfaces or internally.

A transparent material consists of components with a uniform refractive index [18], with the general appearance of one color or any combination leading to a brilliant spectrum of each color. The opposite property of translucency is opacity.

When light encounters a material, it can interact with it in a variety of ways. These interactions depend on the wavelength of the light and the nature of the material. Photons interact with an object through a combination of reflection, absorption, and transmission. Materials transmitting much of the light that falls on them and reflecting little are called optically transparent, a behavior typically exhibited by many liquids, which are characterized by the absence of structural defects (voids, cracks, etc.). Materials that do not transmit light are called opaque. Many of them have a chemical composition that includes centers that may selectively interact with only some of the photons constituting white light. They absorb some portions of the visible spectrum while reflecting others, thus giving rise to color. The attenuation of light of all frequencies and wavelengths is due to the combined mechanisms of absorption and diffusion [19]. 

Many animals that float near the water surface are highly transparent, which results in almost perfect mimetization. Some marine animals, such as jellyfish, have gelatinous bodies, which are composed mainly of water and are largely transparent [20]. Planktonic gelatinous animals are also very transparent. However, transparency is difficult for bodies composed of materials that have refractive indices that differ from water.

Transparency in the air is more difficult to achieve, though a partial example is found in the glass frogs of the South American rainforest, which have translucent skin and pale greenish limbs. Several species of Central American butterflies and many dragonflies and related insects also have wings that are mostly transparent, thus protecting them from predators.

However, with the exception of some tender parts, most plant samples are not sufficiently transparent to allow a view of the internal structures. Conversely, the tissues should be sectioned into slices thin enough to allow for observation by optical means. This is especially true for hard and heavily lignified materials such as wood. 

To obtain a visual indication of the internal part of an object, the reconstruction of numerous thin sections in series has been used. This can be virtually achieved by some tomography techniques or by the direct visualization of thin slices.

An alternative approach was proposed by Spalteholz in 1911, who rendered gross organs transparent by immersion in organic liquids with appropriate refractive indices.

The result was a clear three-dimensional image of the macroscopic structures.

The technique consists of combining the refractive indices of the sample and the medium in which it is incorporated: this leads to the whitening of the organ, which allows the observation of its internal structure. This is achieved by replacing the interstitial fluid sample with a fluid having a similar refractive index. However, the liquids used (formalin, acetone, etc.) are toxic, irritating, and volatile, thus being poorly suitable for anatomic studies.

In 1977, while examining a collection of samples, Hagens considered why plastics were used to embed the samples instead of the opposite, hence stabilizing them from the inside and allowing them to be picked up.

With this in mind, Hagens soaked kidney slices in acetone, followed by its replacement with polymethylmethacrylate under a vacuum. The final sample was transformed into a black, non-transparent mass within an hour. He repeated the experiment using liquid silicone rubber, which had more favorable light-refractive properties. This successful procedure was first reported in a patent [21] and led to the technique that is known now as *plastination* or forced polymer impregnation [22].

Analogously, biologists have shown a similar interest in the anatomy of wood. In this respect, the common technique was that proposed in 1959 by Braun, who, in a long and meticulous study, reconstructed the vessel development of a *Populus* tree from a series of transverse sections, finding that not all run parallel, thus forming a network structure [23].

On this basis, in 1992, S. Fink [15], inspired by Hagen’s work on plastination, proposed this approach to better study the structure of wood at a greater depth.

Observing the structures within the wood in situ at a greater depth requires one to render the wood samples transparent to light through immersion in liquids with a similar refractive index.

To understand this approach, it is first necessary to outline some principles of the behavior of light in wood and other materials: when light passes through a partially transparent material, a part is reflected on the outer and inner surface, a part is absorbed, and a part is refracted. The amount of absorption is due to the coloring of the object: in particular, darker objects absorb more light than brighter ones. In plants, coloration is mainly due to chlorophyll, tannins, and other polyphenols. Therefore, colored samples must undergo a chemical modification by the action of a bleaching solution to become more transparent.

On the other hand, light scattering (i.e., refraction + reflection) occurs in all parts where objects having a different nature (i.e., different refractive index) meet—that is, at the boundaries between the air, vacuole, cell wall, intercellular spaces, cellulose, hemicellulose, lignin, starch, cytoplasm, and chloroplasts.

## 3. Wood Structure

Wood has a complex hierarchical microstructure, characterized by high anisotropy, in which cellulose, hemicellulose, and lignin, its three main components, are interconnected to form a network. 

Cellulose is a polysaccharide consisting of β(1→4) linked D-glucose repeating units with a molecular mass in the range of 10^4^–10^5^ g/mol. Cellulose macromolecular chains mutually interact through H-bonds and van der Waals forces, giving rise to microfibrils, which include both crystalline and amorphous cellulose [24]. The combination of microfibrils forms larger fibrils and lamellae.

Hemicellulose is also a polysaccharide but with a more complex composition that includes xyloglucans, xylans, mannans, and glucomannans. The most important biological role of hemicellulose is its contribution to cell wall strengthening through its interaction with cellulose and lignin [25].

**Figure 2:**
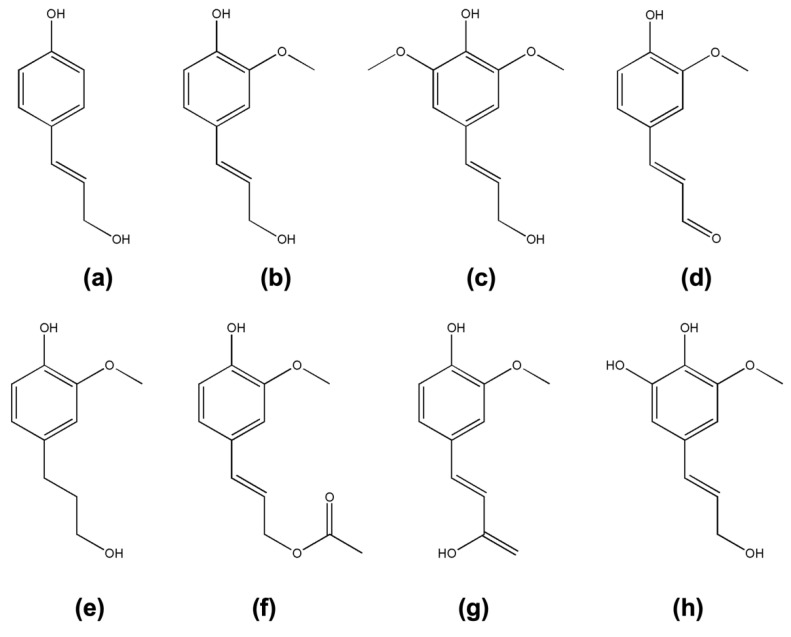
Monolignols typically found in lignin: p-coumaryl alcohol (**a**), coniferyl alcohol (**b**), sinapyl alcohol (**c**), coniferaldehyde (**d**), dihydroconiferyl alcohol (**e**), coniferyl alcohol-9-acetate (**f**), ferulic acid (**g**), and 5-hydroxyconiferyl alcohol (**h**).

Lignins are formed by the polymerization of cinnamyl alcohols (monolignols), which differ in structure depending on the plant type (Figure 2). With a molar mass up to 3 × 10^5^, lignin constitutes an integral part of the cell wall, with chemical bonds to both the above polysaccharides, conferring mechanical strength to the plant. However, it has been shown that the major portion of lignin is more often covalently linked to hemicellulose (i.e., xylan and glucomannan).

While polysaccharides are hydrophilic, lignin is hydrophobic, thus acting as an obstacle to water absorption into the cell wall. As a result, lignin is responsible for the effective conduction of water along the plant vascular tissue.

Wood is composed of numerous cells that are mainly aligned along the longitudinal axis. The lignocellulosic wall of cells contains a primary layer (P) and a secondary layer (S). The cell wall consists of microfibril bundles, containing microfibrils, nanofibrils, elemental fibrils, and macromolecular chains.

**Figure 3:**
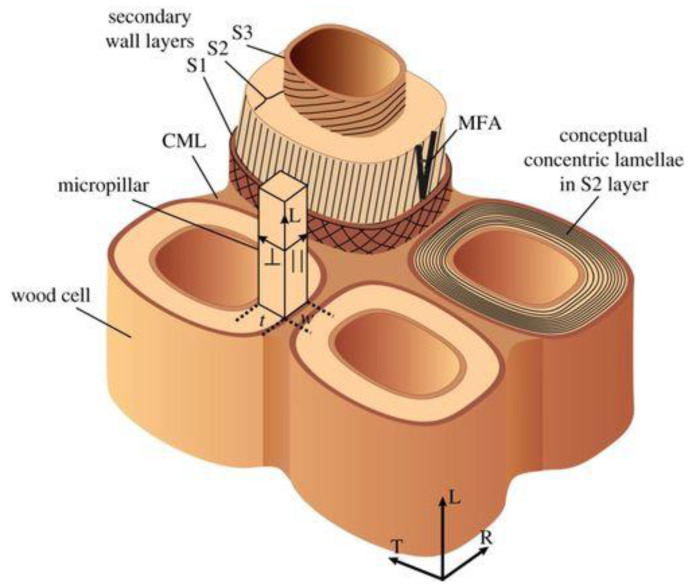
Schematic of cell wall layers within cellular structure of wood. Reprinted from [26] under CC-BY License.

**Figure 4:**
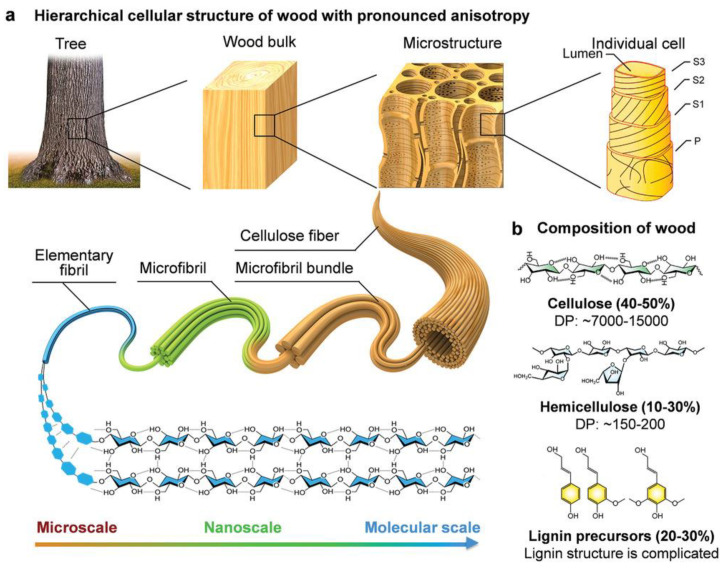
Hierarchical structure of wood and its composition. (**a**) Hierarchical cellular structure of wood is characterized by high anisotropy. The wood is composed of numerous cells that are mainly aligned along the longitudinal axis. The lignocellulosic wall of cells contains a primary layer (P) and a secondary layer (S), which in turn is divided into three sublayers (S1, S2, S3). The cell wall consists of microfibril bundles, which consist of microfibrils, nanofibrils, elemental fibrils, and macromolecular chains. (**b**) The three main components of wood are cellulose, hemicellulose, and lignin. Reprinted with permission from [27], copyright 2021, John Wiley & Sons.

The secondary layer is in turn composed of three sub-layers, S1, S2, and S3, having a thickness of 0.1–0.3, 1–5, and 0.1 μm, respectively (Figure 3 and Figure 4) [26].

The cell walls are bonded by the compound middle lamella (CML), constituted of the primary wall and the middle lamella. Each layer within the secondary cell wall can be considered as a natural fiber-reinforced composite, where the stiff hydrophobic crystalline cellulose microfibrils are closely packed in a hydrophilic matrix of amorphous cellulose, hemicellulose, and lignin. The central and thickest layer, S2, is the most important structural component of the cell wall and provides mechanical support for the tissue.

## 4. Current Methods for Obtaining Transparent Wood

The complexity of the wood structure, its anisotropy, the presence of interfaces among different materials, and light-absorbing chemical groups are responsible for the opacity of wood. However, the application of proper techniques has resulted in modified wood with a high degree of transparency.

For this purpose, two general approaches have been proposed: lignin removal and lignin modification.

### 4.1. Lignin Removal

Lignin removal can be achieved in various chemical conditions ranging from low to high pH values or by exploiting reducing agents.

Alkali treatment has at least a dual effect on lignin. In fact, the linkages among lignin and cellulose or hemicellulose are not stable at high pH values, thus resulting in lignin degradation. In addition, in these conditions, the R-OH groups present in native lignin are converted into R-O^−^, which are much more water-soluble and can be removed more easily from the bulk material. Due to its fundamental importance in the pulping process used in paper manufacturing, there are a plethora of methods to remove lignin by using alkali conditions. Namely, they operate at a relatively high temperature (mostly 140–170 °C) and make use of NaOH or NH_3_ (pure or in solution), often together with sulfur-containing chemicals such as sulfides or sulfites [26].

On the other hand, acidic treatments are carried out at temperatures ranging from approximately 100 to 200 °C and imply the use of strong mineral acids (possible dehydration of polysaccharides is prevented by dilution), with or without organic solvents [26].

However, only some of the above methods can be applied or adapted to obtain TW, a material in which the integrity of polysaccharides should be preserved and the formation of undesirable groups avoided. Furthermore, even if less important, the mechanical properties of the resulting material are also a factor that should be taken into account. 

One of the most effective methods of delignification was reported by Mi et al. [28], who used a prolonged NaClO_2_ bleaching process (up to 24 h) to almost completely remove the lignin (residual lignin: 0.8%) and most of hemicellulose from balsa wood. In addition, nanoscale cellulose fibers with a diameter smaller than the wavelength of visible light were also obtained, thus further increasing the potential transparency of the resulting specimens.

Analogous results were obtained on basswood by He et al. (residual lignin: 0.6%) [29] and by Li et al. (residual lignin: 0.8–1%) [30] by treatment with H_2_O_2_. In all cases, the thickness of the samples (typically in the range of 0.5 to 1.5 mm) played a crucial role, with the best results generally reported for thinner specimens. 

### 4.2. Lignin Modification

In 2017, Li et al. [31] proposed a different approach to prepare TW, in which lignin is not removed from the wood but properly modified only. The authors started from the consideration that lignin removal often implies the use or the formation of toxic substances (including sulfides or chlorine-containing compounds); in addition, because of its structural role, the removal of lignin results in a decrease in the mechanical properties of the final materials.

**Figure 5:**
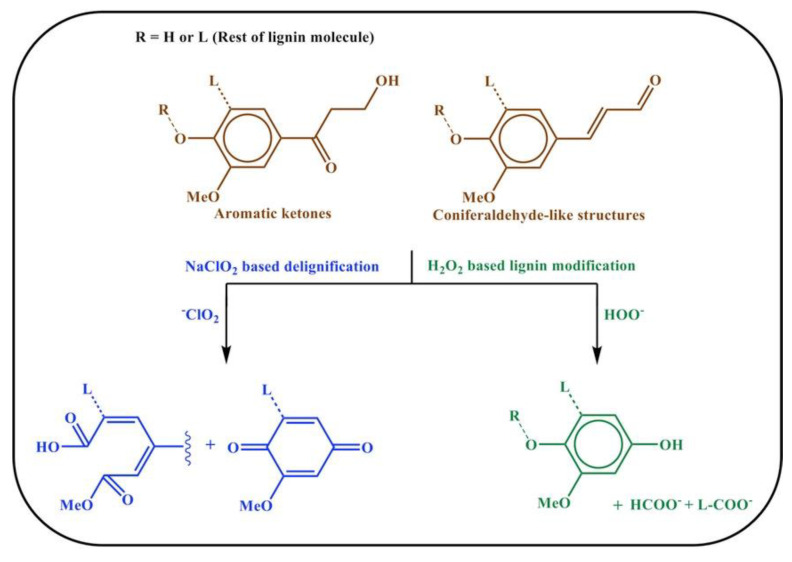
Representative reactions in delignification and lignin modification processes. Reprinted with permission from [31], copyright 2017, John Wiley & Sons.

**Figure 6:**
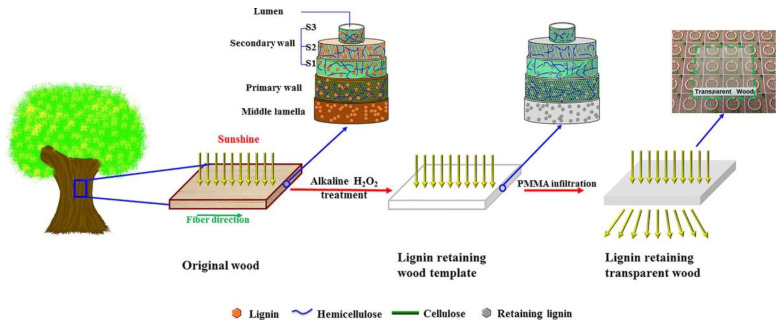
Schematic of process of obtaining transparent wood by lignin modification followed by PMMA infiltration. Reprinted with permission from [31], copyright 2017, John Wiley & Sons.

For these reasons, the authors focused on lignin modification by removing or modifying only the chromophoric groups (Figure 5). After polymethyl methacrylate (PMMA) infiltration, optical properties that were even better than those of TW obtained by lignin removal were found (Figure 6). Moreover, the handling of the wood template was much easier than with delignification.

## 5. Current Methods for Obtaining Transparent Bamboo

Particular consideration should be given to bamboo: while it is commonly considered a type of wood, it is in fact a completely different material.

Bamboo is a subfamily of tall treelike grasses including around 1400 species present in tropical and subtropical to mild temperate regions.

Some bamboo species grow extremely rapidly (30 cm per day). In general, the height of bamboo species is extremely varied, ranging from 10 to 15 cm to more than 40 m. Moreover, it can be grown in a wide range of latitudes. These characteristics make bamboo extremely attractive in the circular economy concept. 

As with wood, bamboo has a largely oriented hierarchy and is composed of the same primary components, namely cellulose, hemicellulose, and lignin. Hence, it is an effective alternative to wood in applications requiring high transparency.

**Figure 7:**
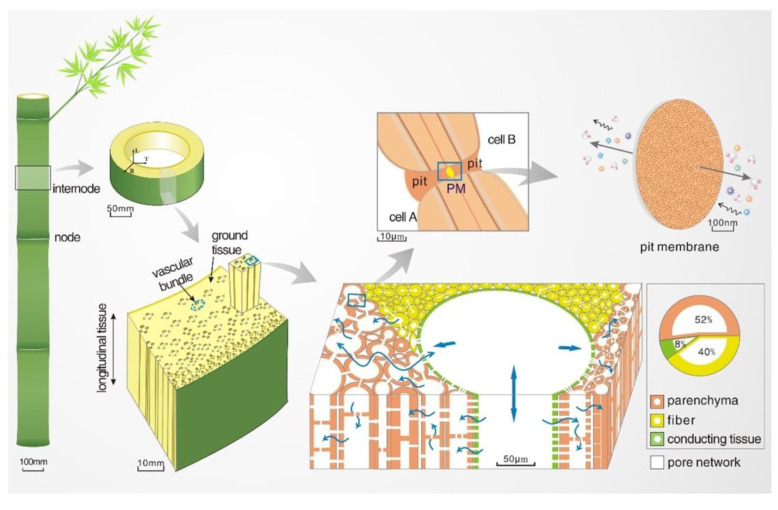
Hierarchical pore network in bamboo moso. Reprinted with permission from [32], copyright 2021, Elsevier.

For the purposes of the present review, a schematic representation of the complex hierarchical pore network in bamboo moso, one of the tallest and more geographically distributed species, is shown in Figure 7.

The preparation of transparent bamboo (TB) consists of two steps. In the first one, the compounds responsible for the bamboo’s color are removed or modified; in the second, the bamboo is impregnated with a monomer or polymer of a suitable refractive index. Indeed, even if removing or modifying the lignin and chromophoric groups is a fundamental step, the delignified or lignin-modified bamboo is still opaque due to the difference among the RIs of the substrate and air. Thus, it is essential to impregnate the bamboo with a transparent resin providing RI matching, to render the resulting composite material transparent.

However, the higher density and lower porosity of bamboo as compared with most of the woods used to produce TW result in increased challenges in TB preparation [33]. 

### 5.1. Lignin Removal

**Figure 8:**
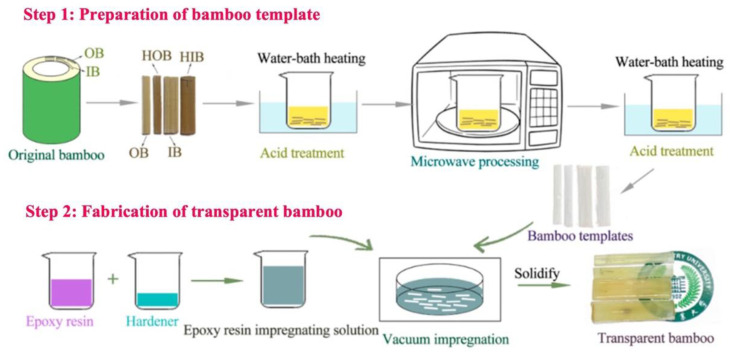
Preparation process of TB by microwave-assisted acidic treatment. Reprinted from [34] under CC-BY license.

Wu et al. have proposed a method involving the use of acetic acid and microwaves for the obtainment of epoxy resin–TB (Figure 8) [34].

The experiments were carried out on two basic units of inner bamboo (IB) and outer bamboo (OB). Part of the IB and OB samples was pre-heated (2 h at 110 °C and 0.2 MPa) to obtain heat-treated inner bamboo (HIB) and heat-treated outer bamboo (HOB), respectively. The main steps of the procedure include (i) the addition of dried bamboo samples (1.1–1.8 mm thick) to a 4 wt.% sodium chlorite water solution containing acetic acid (pH value of 4.6); (ii) heating at 80–90 °C for 2–4 h; (iii) microwave (800 W) application for 5–15 s; (iv) heating in a constant-temperature water tank for 2–3 h; (v) restoration in anhydrous ethanol for 24 h; (vi) desiccation.

**Figure 9:**
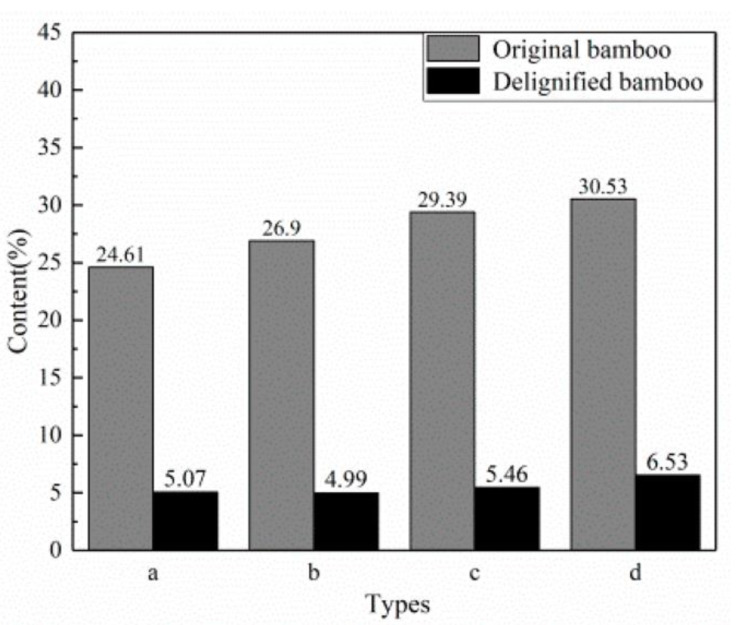
Lignin content of the delignified bamboo samples ((**a**) IB; (**b**) HIB; (**c**) OB; (**d**) HOB). Reprinted from [34] under CC-BY license.

The authors found a significant decrease in lignin content for all types of treated samples (Figure 9), which indicated that the NaClO_2_–CH_3_COOH solution successfully penetrated the bamboo pores, thus removing most of the lignin and resulting in colorless final materials.

### 5.2. Lignin Modification

**Figure 10:**
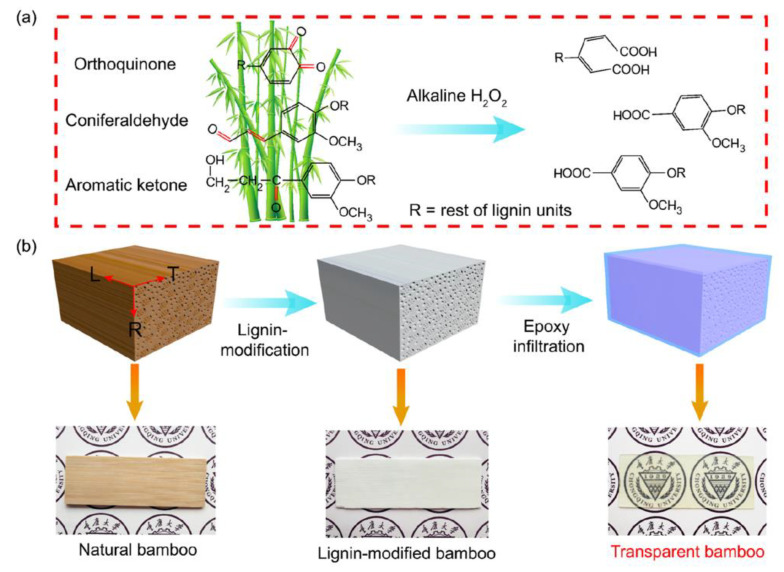
Lignin modification in bamboo moso (**a**) and process of obtaining TB by epoxy infiltration (**b**). Reprinted with permission from [35], copyright 2022, Elsevier.

The application of this approach to bamboo is schematized in Figure 10 [25].

The modification of lignin was achieved by immersing the dried bamboo samples (1.5 mm thick) in an alkaline water solution containing NaOH (3.0 wt.%), Na₂SiO₃ (3.0 wt.%), MgSO_4_ (0.1 wt.%), diethylenetriaminepentaacetic acid (0.1 wt.%), and H_2_O_2_ (4.0 wt.%), heated at 70 °C until they became completely white. The chemicals resulting from lignin modification were then removed by washing with water, followed by ethanol and acetone.

## 6. Recent Applications of Modified/Functionalized Transparent Woods

After delignification, the transparent wood is ready to be modified/functionalized according to the envisaged uses. The treatments that it undergoes are very useful in enhancing the overall performance of the material, usually without significantly affecting the optical properties achieved after the processes.

The modification/functionalization of transparent wood takes advantage of its peculiar micro-, meso-, and macro-porosity, as well as of the chemical structures and compositions of its three main components (i.e., lignin, hemicellulose, and cellulose). According to these characteristics, the employed current methods are focused on three main approaches, namely the infill of the lumen (i.e., of the cavity of a wood cell where free water is held), the modification of the cell wall, or the modification of the interface between the cell wall and lumen. To this aim, it is possible to exploit physical sorption processes, to in situ precipitate metal or inorganic particles from the corresponding salt solutions, to run chemical reactions with reactive monomers, which wood pores and cavities have been previously impregnated with, or to thermally treat the wood’s main components, aiming at promoting phase separation phenomena [17,36]. From a general perspective, all the aforementioned strategies succeed in modifying/functionalizing the transparent wood. In the following, the latest achievements will be described based on the type of envisaged final application of the modified/functionalized material. 

### 6.1. Recent Functional Applications

**Figure 11:**
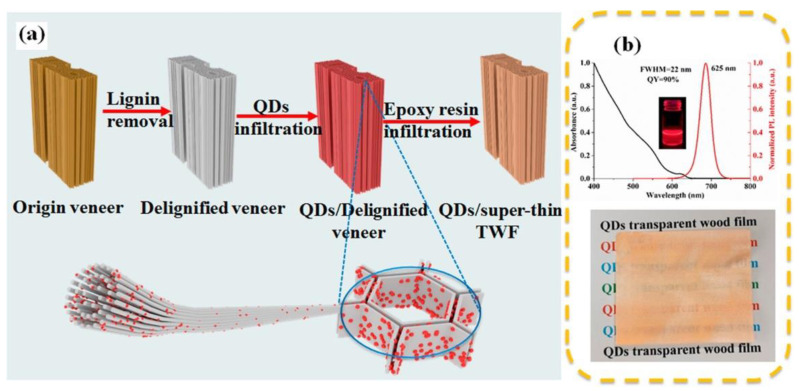
(**a**) Schematic illustration of preparation of QD/superthin transparent wood films (TWFs). (**b**) Luminescence and absorption spectra of the red QD solution (top) and a digital image of the QDs/delignified veneer (bottom). Reprinted with permission from [37], copyright 2022, American Chemical Society.

Superthin transparent wood films (90 μm thickness) for applications in optoelectronics (i.e., photoluminescent films) were recently proposed by Zou and co-workers [37]. For this purpose, a poplar wood veneer was cut into thin slices, delignified, and then treated with solutions of quantum dots (QD) at different concentrations. Finally, the so-obtained material was soaked in a flexible epoxy resin under a vacuum and cured at room temperature for 1 day. A schematic representation of the process is provided in Figure 11.

**Figure 12:**
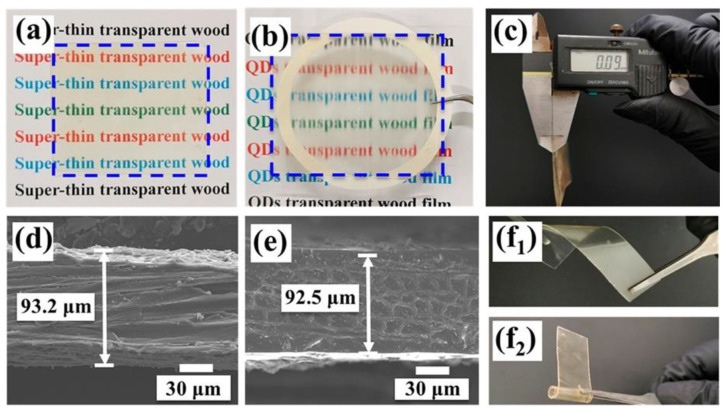
(**a**,**b**) Superthin transparent wood films placed directly on substrate and 10 mm above the substrate, respectively. (**c**) Thickness of the superthin transparent wood films measured with vernier calipers. (**d**,**e**) Thickness of superthin transparent wood films confirmed by SEM. (**f_1_**,**f_2_**) The flexibility of a transparent wood film, rolled in different directions. Reprinted with permission from [37], copyright 2022, American Chemical Society.

**Figure 13:**
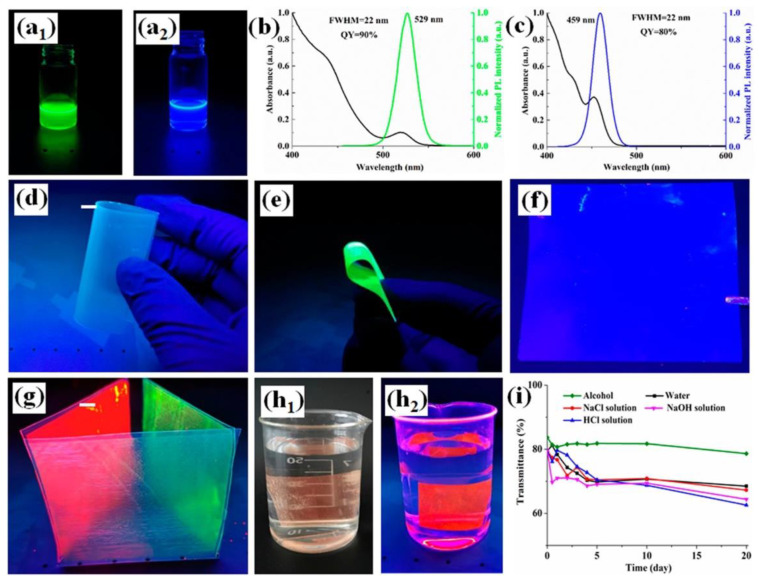
(**a_1_**,**a_2_**) Luminescence of green and blue QD. (**b**,**c**) Absorption and photoluminescence spectra of the green and blue QD solutions. (**d**–**f**) Digital image of a transparent wood film and QD/superthin transparent wood film irradiated by UV light and exhibiting green and blue emission (QD concentration: 0.50 mg/mL). (**g**) Demonstration of light-emitting material using QD/superthin transparent wood films. (**h_1_**,**h_2_**) Digital image of red QD/superthin transparent wood film immersed in water. (**i**) Transmittance of red QD/superthin transparent wood film immersed in harsh environment. Reprinted with permission from [37], copyright 2022, American Chemical Society.

The functionalized thin films exhibited high transparency, as well as good flexibility, as shown in Figure 12. Moreover, the presence of quantum dots (green, blue, or red) in the transparent wood films accounted for the conversion of the incident UV radiation into green, blue (Figure 13), or red light. 

Since the stability of fluorescence is a key issue for the long-term application of the designed photoluminescent materials, some durability tests were performed by placing the films in harsh environments (i.e., aqueous solutions of sodium chloride, sodium hydroxide, or hydrochloric acid, ethanol, and water) for 10 days at ambient temperature, until fluorescence extinction. Besides the behavior in HCl solutions (in which rapid fluorescence decay after 1 day was observed, likely to be attributed to the reaction of the acid with the metals of the QD), the films showed good resistance to the other harsh environments, without losing their light-emitting capabilities (Figure 13). This finding was ascribed to the significant porosity of the delignified wood veneer, which favored the adhesion of the QD to the inner cell wall surfaces, hence protecting the quantum dots and prolonging the photoluminescence.

**Figure 14:**
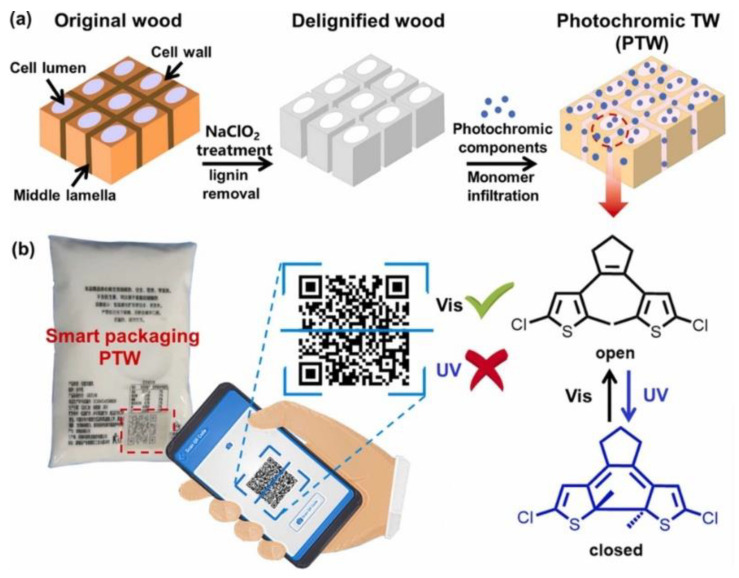
Schematic illustration of the preparation and application of smart photochromic transparent wood (PTW). (**a**) The fabrication process of the PTW. (**b**) Potential smart packaging applications of PTW. Reprinted with permission from [38], copyright 2022, Elsevier.

**Figure 15:**
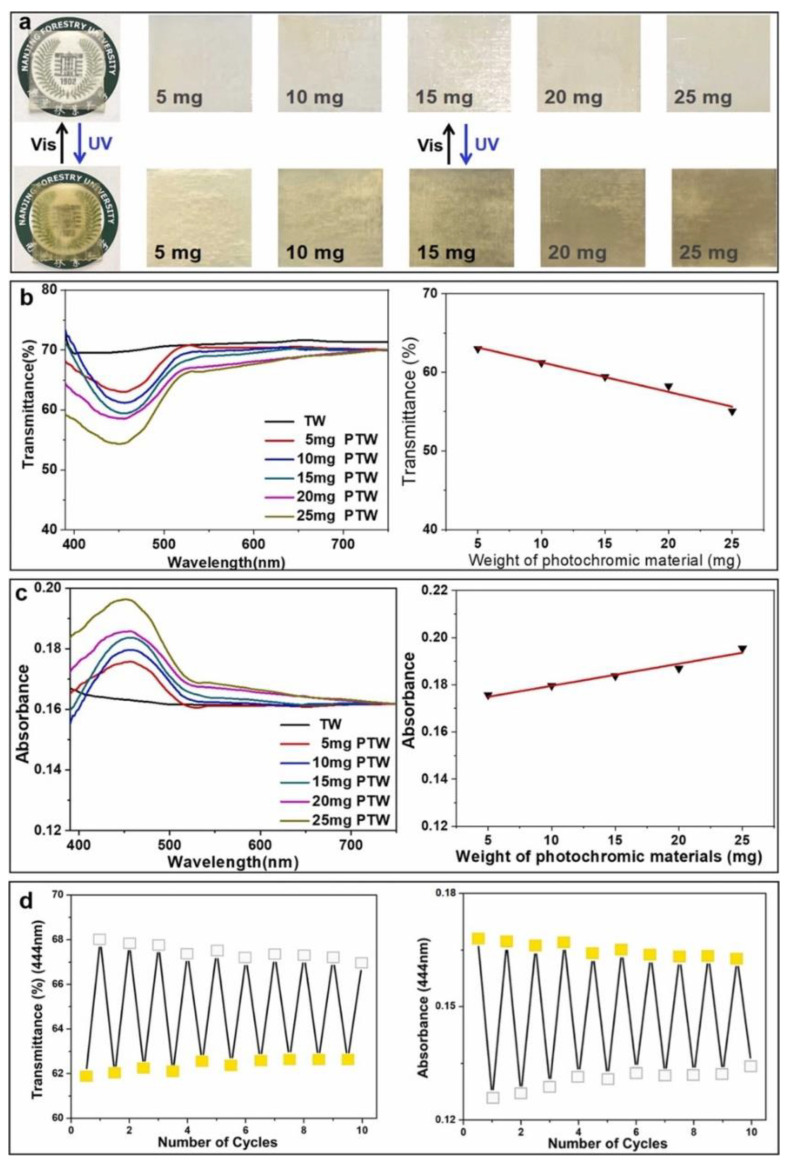
The switchable optical properties of PTW. (**a**) The reversible color changes of the PTW with different concentrations of DTE under UV and visible light irradiation. (**b**) The transmittance and (**c**) absorbance spectra of TW and PTW under irradiation with 254 nm UV light. Here, 5, 10, 15, 20, and 25 mg of DTE correspond to 0.02, 0.04, 0.06, 0.09, and 0.1 wt.% loading in TW. (**d**) The transmittance and absorbance of PTW (25 mg) at 444 nm were collected by a cyclic test through UV (*λ* = 254 nm) and visible light irradiation. Reprinted with permission from [38], copyright 2022, Elsevier.

Another interesting application of transparent wood refers to the design of photochromic materials that can be exploited for smart packaging applications. In this context, Liu and co-workers [38] first delignified poplar wood with NaClO_2_ and then treated it with PMMA and a photochromic dye, namely 1,2-bis(5-chloro-2-methylthiophen-3-yl) cyclopent-1-ene (DTE). The so-obtained photochromic material showed great potential for packaging applications, for which anti-counterfeiting or sensorization functions are required, taking advantage of the possibility of switching its color as a consequence of reversible structural changes involving the DTE rings, which become open or closed upon irradiation with visible or UV light, respectively. A schematic of the overall process, together with a general application of the photochromic effect, is presented in Figure 14. Figure 15 better elucidates the switchable optical features of the obtained photochromic transparent woods.

In addition to the photochromic effects, the prepared materials exhibited outstanding dimensional stability, as well as thermal and mechanical properties suitable for the envisaged applications.

**Figure 16:**
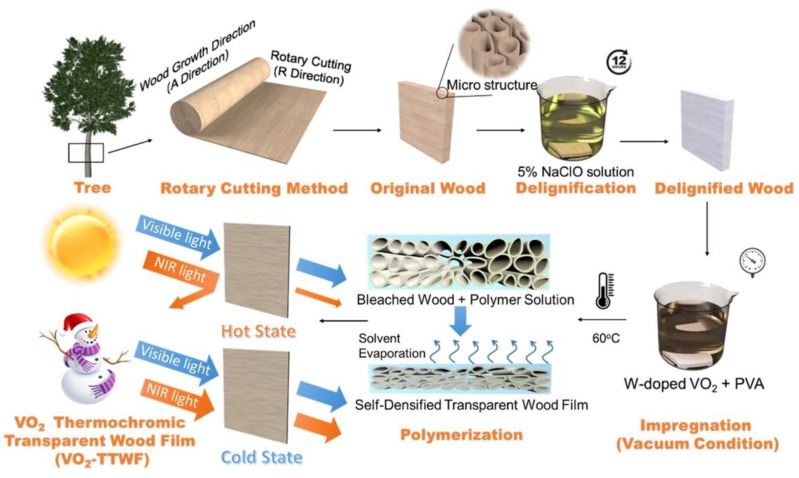
Scheme of the process for obtaining thermochromic transparent wood films containing VO_2_ nanoparticles (VO_2_-TTWF). Reprinted with permission from [39], copyright 2021, American Chemical Society.

Thermochromism is becoming very important in the field of smart glazing materials. In this context, Liu and co-workers [39] designed and prepared multifunctional thermochromic transparent wood films obtained from delignified wood (NaClO treatment). Thermochromism was obtained by vacuum-infiltrating the delignified wood with a suspension of VO_2_ nanoparticles (at different loadings, namely 1, 3, and 6‰) in a poly(vinyl alcohol) solution and subsequent solvent evaporation. The overall process is depicted in Figure 16. Moreover, to achieve hydrophobicity and self-cleaning features, the surfaces of the so-obtained films were further treated with octadecyltrichlorosilane.

**Figure 17:**
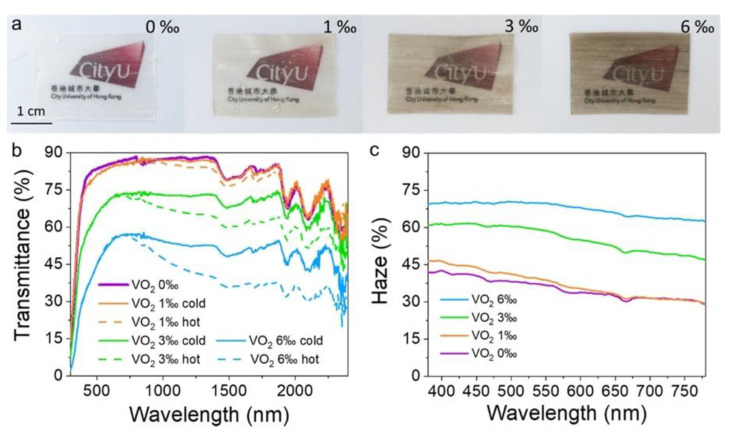
(**a**) Thermochromic transparent wood film (TTWF) with different concentrations of W-doped VO_2_ (0, 1, 3, and 6‰ from left to right); (**b**) temperature-dependent optical transmittance spectra of VO_2_-TTWF; and (**c**) optical transmittance haze of VO_2_-TTWF. VO_2_ X‰ cold and hot refer to transmittance measured after thermal switching from cold to hot state. Reprinted with permission from [39], copyright 2021, American Chemical Society.

**Figure 18:**
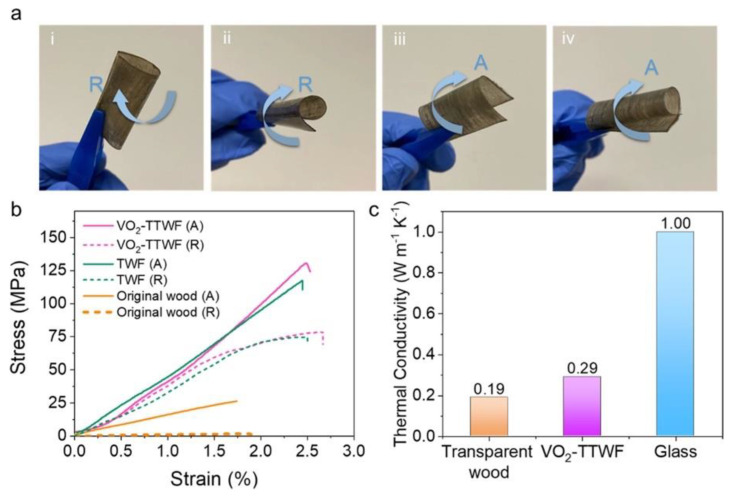
(**a**) VO_2_-TTWF was folded and rolled in both A (axial) and R (radial) directions, as shown in the images i–iv. (**b**) Stress–strain curves of VO_2_-TTWF, TWF, and original wood in A and R directions. (**c**) Comparison of thermal conductivity of VO_2_-TTWF, transparent wood, and glass. Reprinted with permission from [39], copyright 2021, American Chemical Society.

The obtained material exhibited 70% haze and high temperature-dependent transmittance within the visible wavelength range (Figure 17), superhydrophobicity (the static water contact angle was approximately 122°), low thermal conductivity (approximately 0.29 W m^−1^ K^−1^, measured in the transverse direction with respect to the wood fiber growth), and improved mechanical behavior, with respect to both pristine and delignified wood (Figure 18). All these findings support the potential of the designed films for smart glazing applications.

**Figure 19:**
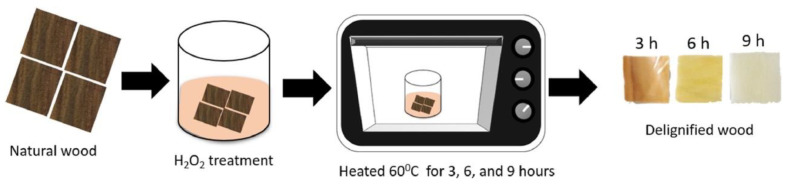
Scheme of the preparation of transparent wood. Reprinted with permission from [40], copyright 2022, Elsevier.

Muhammad and co-workers demonstrated that functionalized transparent wood is able to act as an efficient X-ray shielding material [40]. For this purpose, sonokeling wood was first delignified (by means of H_2_O_2_ treatment carried out at 60 °C for 3, 6, or 9 h) and then treated with a poly(vinyl alcohol)/gelatin solution, containing 0.1, 0.3, or 0.5 g of BaCO_3_ as an X-ray shielding product. After drying, the X-ray shielding capability of the obtained transparent wood was tested for different photon energies (namely, 55, 66, 77, and 90 keV). A schematic representation of the fabrication of functionalized transparent wood is depicted in Figure 19.

**Figure 20:**
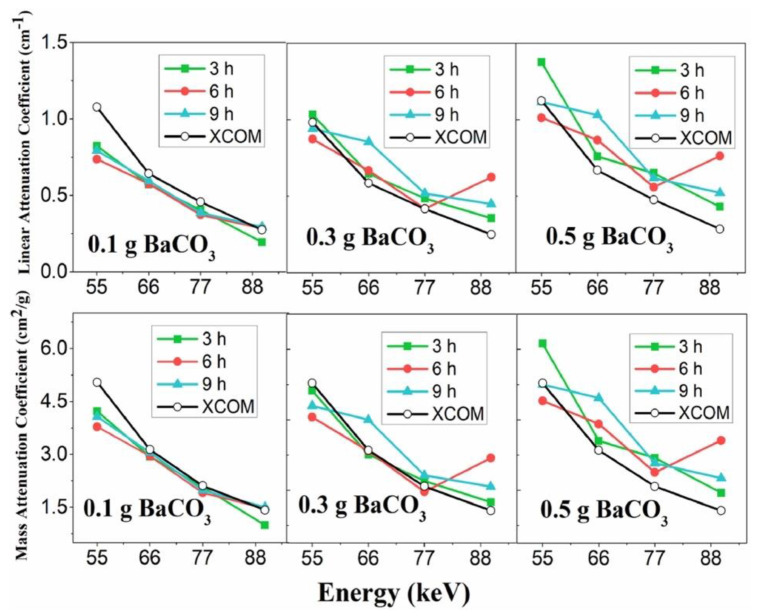
The experimental values of linear attenuation coefficient (µ) and mass attenuation coefficient (µ_m_) for different delignification times and BaCO_3_ content (filled symbol indicated by 3, 6, and 9 h). For comparison, the theoretical calculation (XCOM database) from the National Institute of Science and Technology (India) (open symbol) is included. Reprinted with permission from [40], copyright 2022, Elsevier.

As assessed by the X-ray shielding tests (Figure 20), high linear attenuation coefficients were measured for all the photon sources investigated. Moreover, the mass attenuation coefficients were found to linearly increase with increasing BaCO_3_ loading.

**Figure 21:**
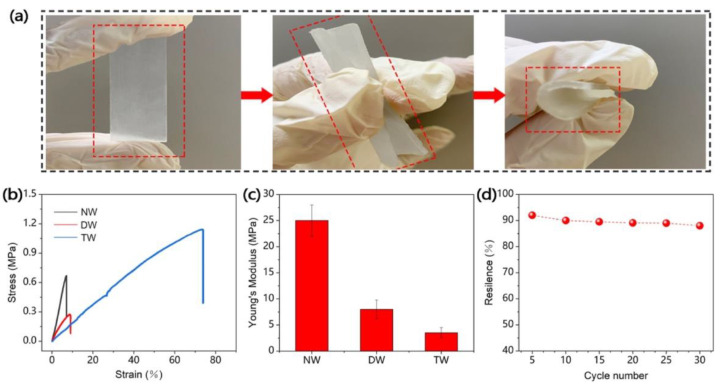
Mechanical properties of transparent wood. (**a**) Transparent wood becomes highly flexible upon bending. (**b**) Typical stress–strain curves of natural (pristine) wood (NW), delignified wood (DW), and transparent wood (TW). (**c**) Young’s modulus of NW, DW, and TW. (**d**) Resilience of the TW at different cycle numbers measured under 40% strain. Reprinted permission from [41], under CC-BY license.

**Figure 22:**
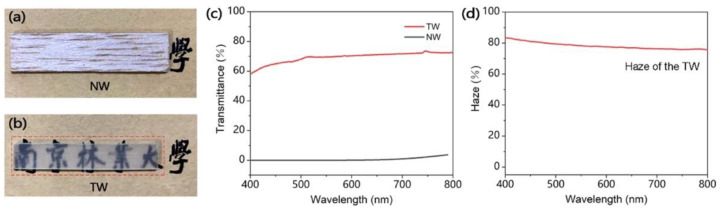
Comparison of optical properties between natural (pristine) wood (NW) and transparent wood (TW). (**a**,**b**) Digital images of the NW and TW laid on the same pattern. (**c**) Optical transmittance of the NW and TW. (**d**) Optical haze of the TW. Reprinted permission from [41], under CC-BY license.

**Figure 23:**
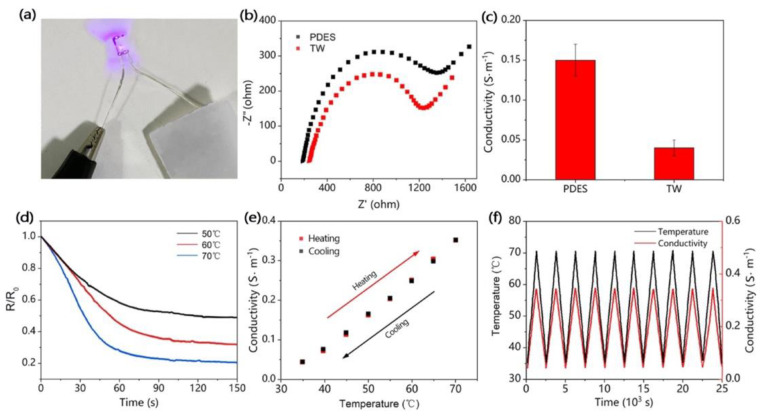
Electrical properties and temperature sensing performance of transparent wood (TW). (**a**) Digital image to demonstrate that the TW can illuminate an LED bulb. (**b**) Electrochemical impedance spectroscopy (EIS) plots of the deep eutectic solvent polymer (PDES) and TW. (**c**) Plots of calculated conductivity values for PDES and TW. (**d**) The resistance change curve of TW at 50, 60, and 70 °C shows the dependence of the TW resistance change on temperature. (**e**) The dependence of the conductivity change of TW on the temperature shows an approximately linear relationship within one cycle of heating and cooling. (**f**) Change in conductivity of TW in 10 temperature change cycles. During the electrical testing, the applied voltage in all the electrical tests was 0.5 V. Reprinted with permission from [41], under CC-BY license.

Yang and co-workers [41] succeeded in obtaining a flexible transparent wood, suitable for applications in the field of sensors and flexible electronics, derived from the delignification of balsa wood (treatment with NaClO_2_ and subsequent bleaching with H_2_O_2_) and infiltrated with a polymerizable deep eutectic solvent comprising acrylic acid and choline chloride. Besides remarkable mechanical behavior (in the form of high flexibility, stretch resilience, and stretchability, as shown in Figure 21) and acceptable optical properties (transmittance and haze were beyond 60% and 70%, respectively, within the visible wavelength range; see Figure 22), the treatment with the deep eutectic solvent led to interesting electrical conductivity and temperature sensing features (Figure 23), which were attributed to the free movement of positive and negative ions provided by choline chloride in the deep eutectic mixture. Moreover, during repeated heating–cooling cycles, the electrical signal of the modified transparent wood was very stable and repeatable, hence indicating its great potential for the design of temperature sensors. 

### 6.2. Recent Energy-Saving Applications

**Figure 24:**
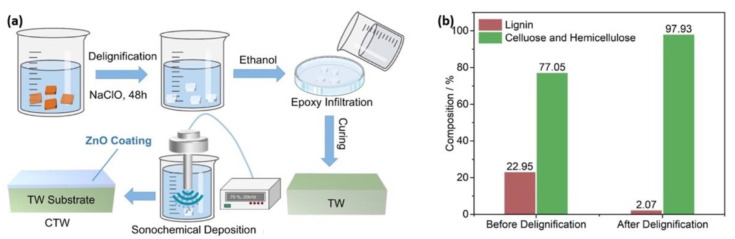
(**a**) Schematic manufacturing procedures of coated transparent wood (CTW); (**b**) chemical compositions of balsa wood before and after delignification. Reprinted with permission from [42], copyright 2022, Elsevier.

Since its discovery, transparent wood has been exploited in the building sector as a tough alternative to glass; over time, the interest in transparent wood for civil engineering applications has been focused on novel uses as a passive radiative cooling material with improved energy efficiency. In this context, Hu and co-workers [42] utilized a sonochemical treatment for the functionalization of transparent wood (namely balsa wood) with a zinc oxide coating. The overall process is depicted in Figure 24. First, the wood was delignified with a NaClO solution and then repeatedly washed with ethanol and distilled water and finally stored in ethanol. Next, the delignified wood was dipped into the epoxy system and subsequently transferred into a vacuum dryer and degassed, hence allowing for the resin infiltration process and its curing at room temperature for at least 36 h. The cured transparent wood/epoxy system was finally sonochemically treated to deposit a ZnO coating on its surface, washed with ethanol and distilled water, and finally dried. 

**Figure 25:**
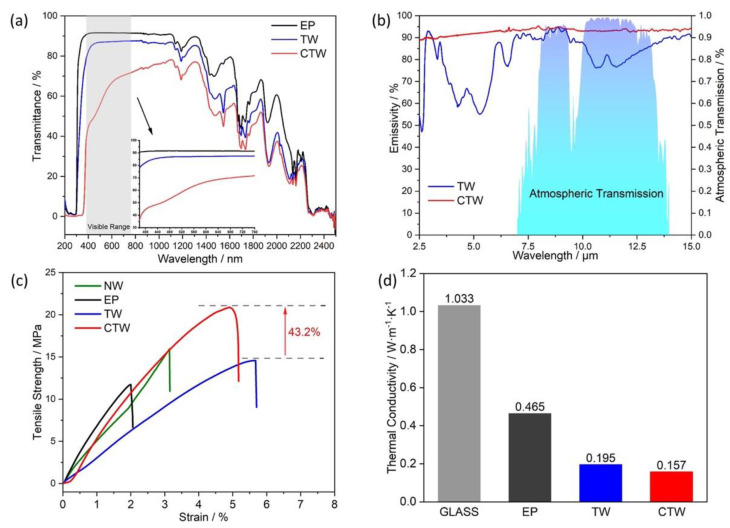
(**a**) Transmittance vs. wavelength curves of cured epoxy resin (EP), uncoated transparent wood (TW), and ZnO-coated transparent wood (CTW); (**b**) emissivity spectrum of TW and CTW within the long-wavelength infrared region; (**c**) stress–strain curves of natural wood (NW), EP, TW, and CTW; (**d**) thermal conductivity of glass, EP, TW, and CTW. Reprinted with permission from [42], copyright 2022, Elsevier.

Figure 25 compares the overall optical and mechanical performance of the coated transparent wood (CTW) with that of the cured epoxy resin (EP) and of the uncoated transparent wood (TW). CTW exhibited acceptable transmittance in the visible region (approximately 66%, measured on 360-nm-thick films), good solar reflectance, outstanding thermal insulation (its thermal conductivity was as low as 0.157 W m^−1^K^−1^), good mechanical behavior (required for glazing materials), and high emissivity (approximately 90% within the long-wavelength infrared region). 

**Figure 26:**
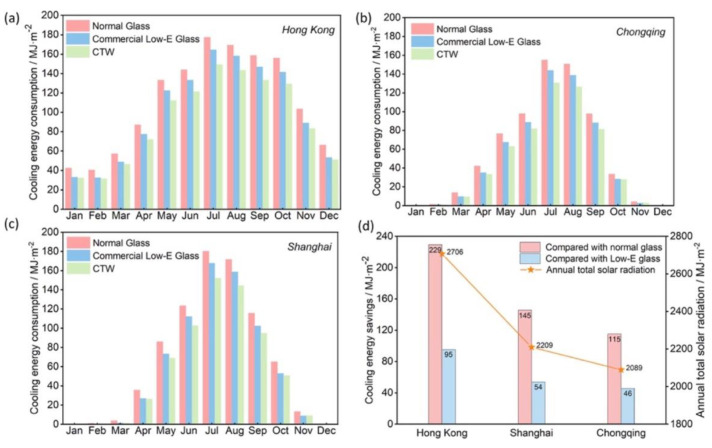
Monthly cooling energy consumption in Hong Kong (**a**), Chongqing (**b**), Shanghai (**c**); annual cooling energy saving compared with the other two types of glazing materials, i.e., standard glass and low-emissivity (Low-E) glass, and annual total solar radiation for the three cities considered (**d**). Reprinted with permission from [42], copyright 2022, Elsevier.

Finally, simulations confirmed the cooling energy-saving potential of ZnO-coated transparent wood with respect to standard commercial glass and to Low-E glass glazing, in three large Chinese cities (i.e., Hongkong, Shanghai, and Chongqing), as shown in Figure 26. 

**Figure 27:**
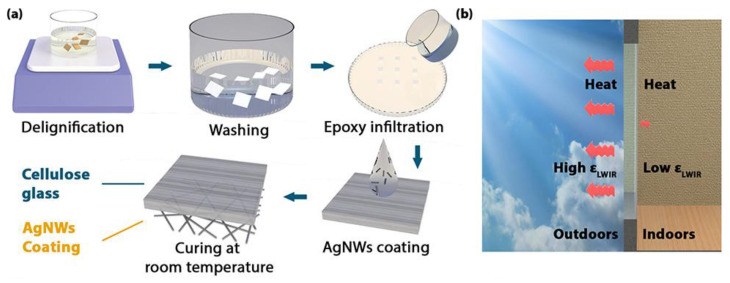
(**a**) Schematic manufacturing procedures of the radiative cooling transparent bamboo; (**b**) schematic demonstration of the working mechanism of the radiative cooling transparent bamboo. AgNWs = silver nanowires; ε_LWIR_ = long-wavelength infrared emissivity. Reprinted with permission from [43], copyright 2021, American Chemical Society.

Aiming at obtaining a transparent wood with improved radiative cooling, hence suitable for energy-saving building applications, Zhou et al. [43] delignified bamboo that was subsequently infiltrated with an epoxy system and cured. Afterwards, silver nanowire coatings (composed of up to 18 layers) were deposited on one side only of the transparent wood, using a spin coater (Figure 27). In this way, it was possible to create a surface coated with silver nanowires, with very low (i.e., 0.3) long-wavelength infrared emissivity, placed indoors, and an uncoated surface, with long-wavelength infrared emissivity of 0.95, placed outdoors, and able to stimulate radiative cooling, through the emission of the heat absorbed by the transparent wood to the outer environment. 

**Figure 28:**
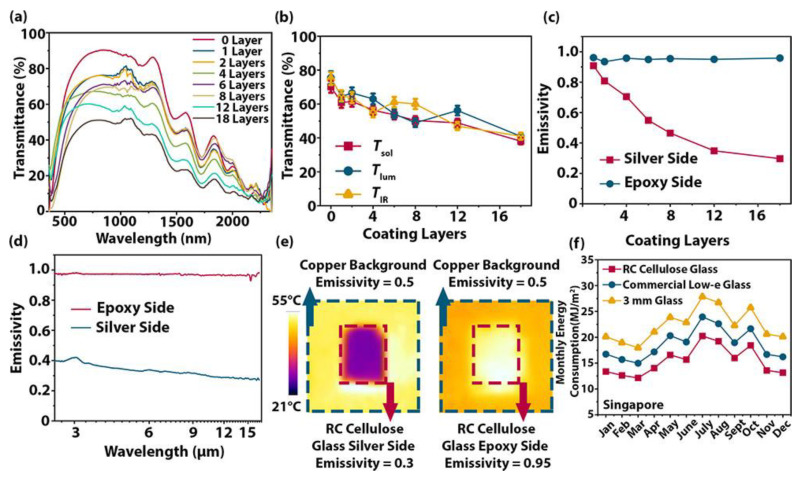
(**a**) UV–vis–NIR spectra of the radiative cooling transparent bamboo with various numbers of silver nanowire layers; (**b**) integrated *T*_sol_ (solar transmittance), *T*_lum_ (luminous transmittance), and *T*_IR_ (infrared transmittance) values of the radiative cooling transparent bamboo as a function of the number of silver nanowire layers; (**c**) emissivity of the radiative cooling transparent bamboo as a function of the number of silver nanowire layers; (**d**) long-wavelength infrared emissivity spectrum of the two sides (one coated with 18 layers) of the radiative cooling transparent bamboo; (**e**) IR image of both sides (one coated with 18 layers) at 50 °C, using a 0.5 emissivity copper block as the background; (**f**) simulated monthly energy consumption in Singapore when 3 mm conventional glass, commercial low-E glass, and radiative cooling transparent bamboo are used. Reprinted with permission from [43], copyright 2021, American Chemical Society.

As expected, the transmittance and the emissivity were found to decrease upon increasing the number of deposited silver nanowire layers (Figure 28). Moreover, as shown in Figure 28f, simulations carried out for Singapore confirmed the energy-saving potential of the radiative cooling transparent bamboo with respect to standard commercial glass and to Low-E glass glazing, with an improvement of up to 89% on a yearly basis. 

**Figure 29:**
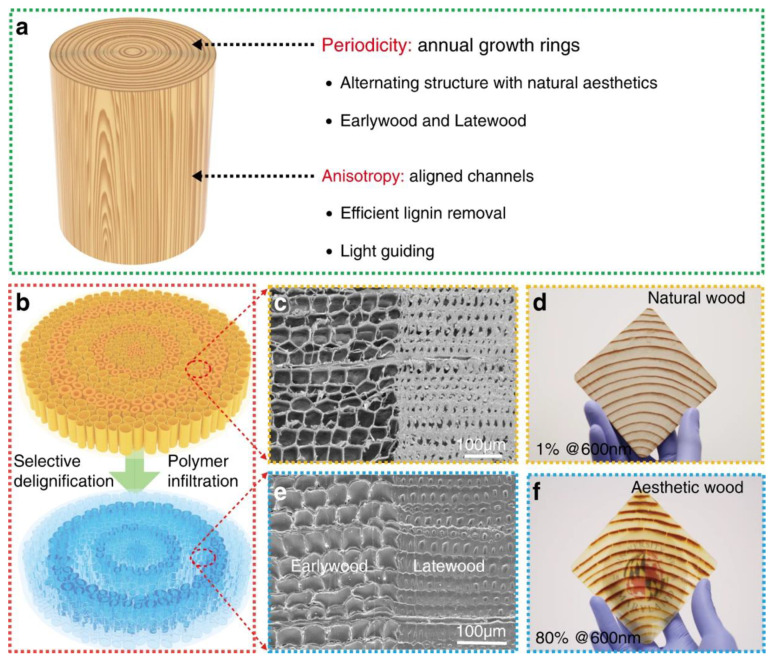
(**a**) An indication of the design that combines the periodicity (annual growth rings) with the anisotropy (aligned channels) of wood to produce a new type of transparent wood composite. (**b**) Schematic displaying the procedures for fabricating aesthetic wood (aesthetic wood-R) from natural Douglas fir wood with vertically aligned cells and annual growth rings after fast, spatially selective delignification and polymer infiltration. (**c**,**e**) Typical cross-sectional SEM images of natural wood and dense aesthetic wood-R microstructures after polymer infiltration (there is a sharp boundary between earlywood—EW and latewood—LW). (**d**,**f**) Photos showing a large piece of aesthetic wood-R (86 × 86 × 2 mm^3^) with preserved wood patterns and high average transparency (80% transmittance at 600 nm) derived from Douglas fir. Reprinted from [44] under CC-BY license.

Energy-saving requirements were recently combined with an aesthetic function by Mi and co-workers [44], who demonstrated the suitability and scalability of aesthetic transparent wood, i.e., a material that undergoes a process involving the spatially selective removal of lignin and subsequent resin (e.g., epoxy) infiltration, for the design of energy-efficient building applications. More specifically, as shown in Figure 29, the particular delignification process allowed for the preservation of the original growth ring patterns of the wood, giving rise to the formation of two types of aesthetic wood, one (wood-R) bearing aligned microchannels perpendicular to the wood plane, and the other (wood-L) showing aligned microchannels parallel to the wood plane.

**Figure 30:**
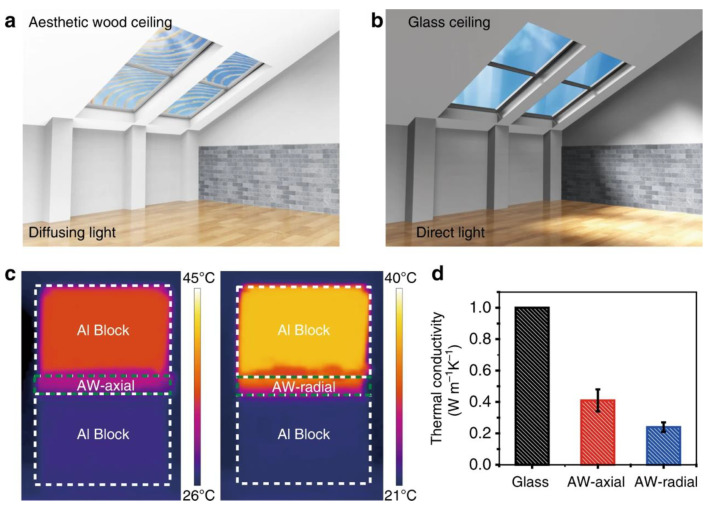
(**a**,**b**) The schematic shows the light distribution and aesthetic appeal inside a building when applying the aesthetic wood (abbreviated as AW in the (**d**) ceiling), compared with the glass ceiling. (**c**) IR images of aesthetic wood with temperature distributions in the axial (AW-axial heat transfer direction is parallel to the aligned wood microchannels) and radial (AW-radial heat transfer direction is perpendicular to the aligned wood microchannels) directions. (**d**) Thermal conductivities of glass [45], axial (AW-axial) and radial (AW-radial) direction of aesthetic wood. Error bars represent standard deviation. Reprinted from [44] under CC-BY license.

Moreover, besides the excellent optical properties (i.e., around 80% transmittance at 650 nm and 93% optical haze, this latter being important for creating uniform indoor light distribution) and low thermal conductivity (0.24 W m^−1^ K^−1^), the aesthetic wood showed very high mechanical strength (approximately 92 MPa), as well as remarkable toughness (2.73 MJ m^−2^): all these features support its use for building glazing (Figure 30).

An interesting approach, which takes advantage of the in situ polymerization of an epoxy-based dynamic covalent polymer, i.e., exhibiting a thermally induced exchangeable dynamic covalently cross-linked network, was recently proposed by Wang and co-workers on transparent wood suitable for building applications [46]. In particular, a quick epoxy-thiol click reaction was exploited for the curing of the selected epoxy system (at 120 °C for 2 h), previously vacuum-infiltrated in balsa wood. The so-obtained material exhibited low thermal conductivity values (between 0.29 and 0.30 W m^−^^1^ K^−^^1^), as well as high haze and transmittance (respectively, around 95 and 60%, measured on 2-mm-thick samples of transparent wood). Moreover, the transparent wood showed high shape manipulation ability, comprising shape memory, shape-editable, and shape recovery features attributable to the solid-state, thermally induced plasticity of the epoxy-based dynamic covalent structure of the employed epoxy system. All these features suggest the potential suitability of the designed material for smart, shape memory building glazing applications.

**Figure 31:**
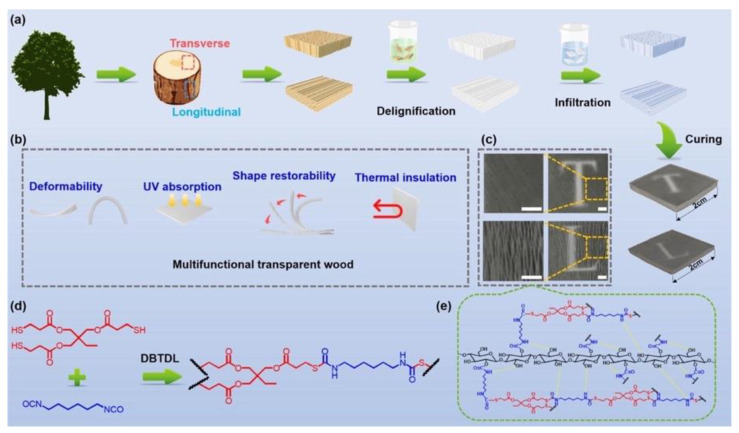
Synthesis process, multiple functions, macroscopic images, reaction scheme, and bonding mechanism of TWs. (**a**) Synthesis process of transparent wood. (**b**) Deformability, UV absorption, shape restorability, and thermal insulation functions of transparent wood. (**c**) Digital images of transparent wood at different magnification times (the inserted ruler indicates a length of 2 mm). (**d**,**e**) Reaction of vitrimers and bonding mechanism between cellulose and vitrimers. DBTDL = Dibutyltin dilaurate. Reprinted with permission from [47], copyright 2022, Elsevier.

In a further research effort, the same group [47] demonstrated the high potential of delignified balsa wood, vacuum-infiltrated by stimuli-responsive vitrimers (bearing exchangeable dynamic thiocarbamate links) and cured at a low temperature (not exceeding 60 °C) using a solvent-free thiol-isocyanate click-reaction approach. The overall process is schematized in Figure 31.

**Figure 32:**
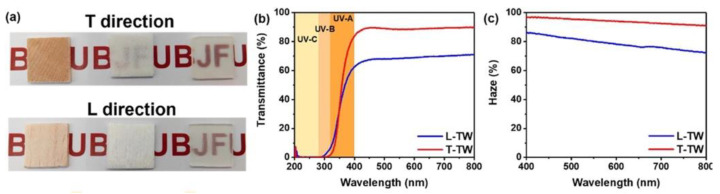
Optical properties of the transparent wood (TW). (**a**) From left to right, transverse (T direction) and longitudinal (L direction) digital images of wood, delignified wood, and transparent wood (thickness = 2 mm). (**b**,**c**) Total transmittance and haze of transverse (T-TW) and longitudinal (L-TW) transparent wood (thickness = 2 mm). Reprinted with permission from [47], copyright 2022, Elsevier.

**Figure 33:**
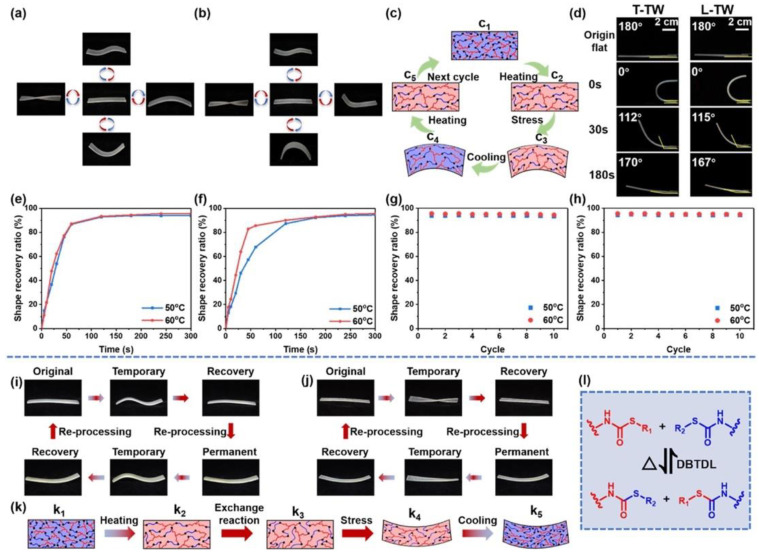
Shape deformation properties of transverse (T-TW, (**a**)) and longitudinal (L-TW, (**b**)) transparent wood. (**c**) Shape memory mechanism of transparent wood. (**d**) Shape restorability of T-TW and L-TW at 50 °C. (**e**,**f**) Shape recovery curves of transverse (T-TW) and longitudinal (L-TW) transparent wood at 50 °C and 60 °C. (**g**,**h**) Shape recovery ratio of T-TW and L-TW within 10 cycles at 50 °C and 60 °C. (**i**,**j**) Shape reprocessing performance of T-TW and L-TW. (**k**) Shape reprocessing mechanism of TW. (**l**) Exchange reaction mechanism of vitrimers above topology freezing transition temperature (Tv). DBTDL = Dibutyltin dilaurate. Reprinted with permission from [47], copyright 2022, Elsevier.

The so-obtained infiltrated transparent wood exhibited outstanding optical properties, with both haze and transverse transmittance of around 90%, as presented in Figure 32. Moreover, its thermal conductivity was as low as 0.30 W m^−^^1^ K^−^^1^, and it possessed very good mechanical features (tensile and flexural strength of around 37 and 49 MPa, respectively), thus suggesting its suitability for applications in the building sector as a smart, energy-efficient material. Finally, as shown in Figure 33, the infiltration of the vitrimer system accounted for an appealing shape manipulation, with rapid recovery to approximately 170° within 180 s.

### 6.3. Recent Flame-Retardant Applications of Transparent Wood 

As with most polymer systems, if not inherently flame-retardant, wood (and transparent wood) quite easily burns when exposed to an irradiative heat source or a direct flame. To overcome this issue, some strategies were recently proposed.

Zhang and co-workers [48] incorporated MXene^®^ nanosheets (at 1, 2, and 3 wt.% loading) into poly(vinyl alcohol) and then infiltrated the dispersion into delignified balsa wood (NaClO_2_ treatment). The so-obtained material exhibited enhanced tensile strength, good transparency (even at the highest MXene loading), low thermal conductivity (equal to 0.31 W m^−^^1^ K^−^^1^), and improved UV resistance with respect to the delignified balsa wood. Further, as assessed by microscale combustion calorimetry tests, the incorporation of poly(vinyl alcohol) containing 2 wt.% of MXene nanosheets accounted for a significant decrease in both the peak heat release rate and total heat release with respect to the delignified balsa wood (by approximately 9 and 22%, respectively).

**Figure 34:**
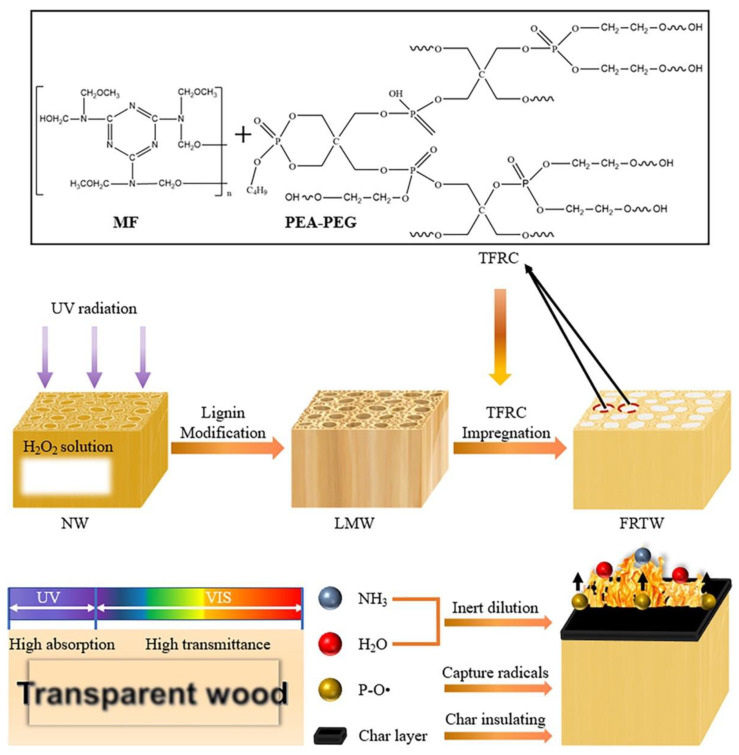
Schematic illustration of the preparation of flame-retardant transparent wood (FRTW), together with the demonstration of its optical and flame-retardant properties. NW = natural (pristine) wood; LMW = lignin-modified wood; MF = melamine–formaldehyde resin; PEA-PEG = flame-retardant product obtained by reacting poly(ethylene glycol) with cyclic phosphate ester; TFRC = transparent flame-retardant resin system. Reprinted with permission from [49], copyright 2022, John Wiley & Sons.

**Figure 35:**
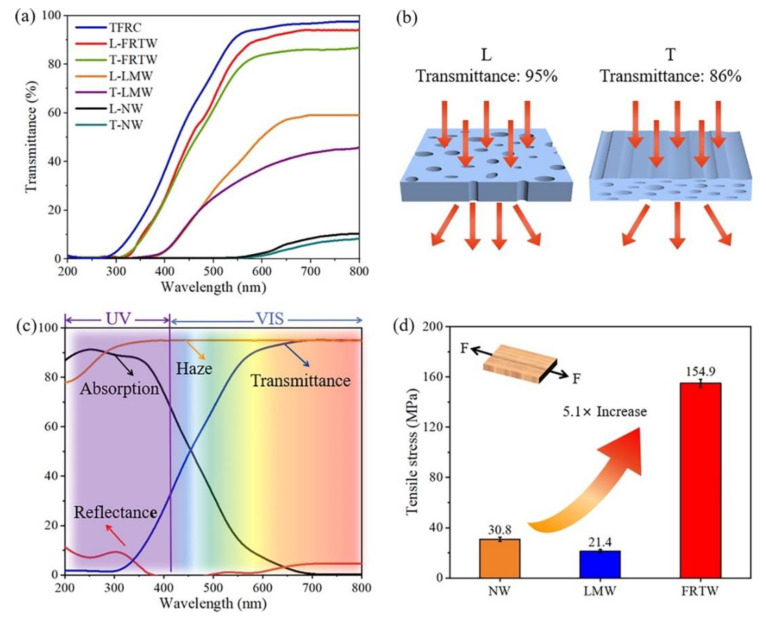
Optical and mechanical properties of wood samples. (**a**) The optical transmittance of natural (pristine) wood (NW), lignin-modified wood (LMW), flame-retardant transparent wood (FRTW), and transparent flame-retardant cured resin system (TFRC). (**b**) Schematic illustration of light propagation through the FRTW in the L (parallel to the wood growth direction) and T (perpendicular to the wood growth direction) modes. (**c**) Absorption, haze, reflectance, and transmittance of L-FRTW. (**d**) Tensile stress of the NW, LMW, and FRTW samples. Reprinted with permission from [49], copyright 2022, John Wiley & Sons.

**Figure 36:**
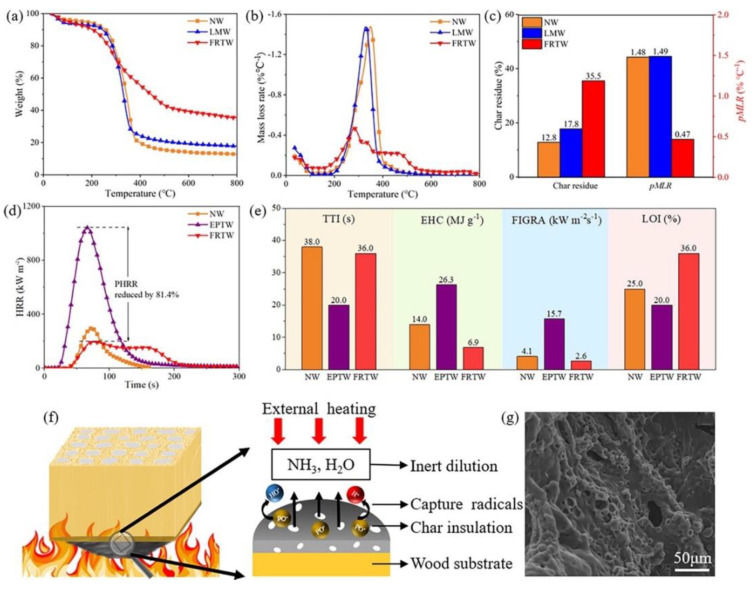
(**a**) Thermogravimetric and (**b**) derivative thermogravimetric (DTG) curves of natural (pristine) wood (NW), lignin-modified wood (LMW), and flame-retardant transparent wood (FRTW). (**c**) Comparison of char residue and peak mass loss rate (pMLR) for NW, LMW, and FRTW. (**d**) Heat release rate (HRR) curves of NW, EPTW, and FRTW, which highlight the decrease in peak heat release rate (PHRR). Comparison of (**e**) time to ignition (TTI), effective heat of combustion (EHC), fire growth rate index (FIGRA), and limiting oxygen index (LOI) for NW, EPTW, and FRTW. (**f**) Flame-retardant mechanism of FRTW under external heating. (**g**) A typical SEM image of the porous char residues of FRTW. EPTW = transparent balsa wood impregnated with a standard epoxy system. Reprinted with permission from [49], copyright 2022, John Wiley & Sons.

Fan et al. [49] exploited UV radiation to oxidize the chromophores present in the lignin of balsa wood in the presence of hydrogen peroxide. Then, a P-containing flame retardant, obtained from the reaction of poly(ethylene glycol) with a cyclic phosphate ester, was incorporated into a melamine–formaldehyde resin and utilized to impregnate the previously obtained transparent wood. Figure 34 schematizes the overall process. After curing, the flame-retardant material showed high haze and transparency (with transmittance of around 95% within the visible light range). Moreover, the modification of balsa wood accounted for a significant increase in tensile strength (Figure 35). Finally, as assessed by thermogravimetric analyses and flammability (limiting oxygen index) and cone calorimetry tests, the flame-retardant transparent wood exhibited enhanced thermal stability and fire behavior (Figure 36), evidenced by the increase in char residue and limiting oxygen index (which reached 36%) and the decrease in the peak heat release rate (PHRR, −81%), effective heat of combustion (EHC, approximately −74%), peak mass loss rate (pMLR, −69%), and fire growth rate index (FIGRA, −83%), with respect to the transparent wood impregnated with a standard epoxy system. All these findings support the suitability of the designed flame-retardant transparent wood for optical and building purposes.

**Figure 37:**
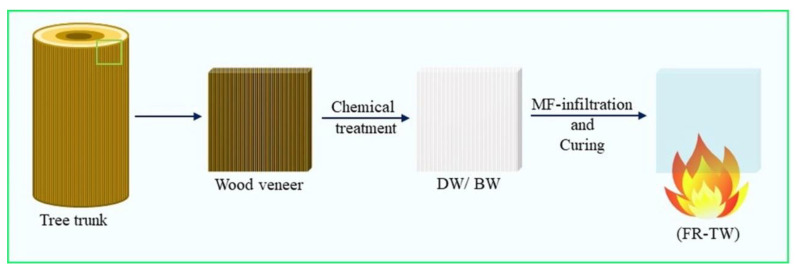
Schematic illustration of fire-retardant transparent wood (FR-TW) fabrication based on melamine formaldehyde, MF. DW is delignified wood where most lignin is removed, and BW is bleached wood where lignin chromophores are modified and a small amount of lignin removed. Reprinted from [50] under CC-BY license.

A similar approach was recently proposed by Samanta et al. [50], who subjected a balsa wood veneer and birch wood to either delignification (by means of NaClO_2_) or bleaching (to modify lignin chromophores); the resulting materials were infiltrated by a water-soluble and flame-retardant melamine–formaldehyde resin and cured. A schematic of the overall process is presented in Figure 37.

**Figure 38:**
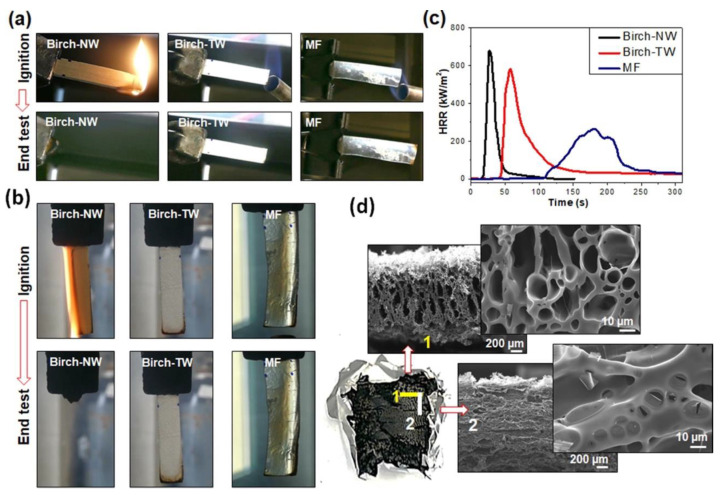
Flame spread test snapshots (**a**) in horizontal, (**b**) in vertical configuration, and (**c**) heat release rate vs. time curves for pristine birch wood (Birch-NW), flame-retardant birch wood (Birch-TW), and cured melamine–formaldehyde resin (MF); (**d**) cross-section SEM micrographs of the residues of Birch-TW in two perpendicularly oriented planes. Reprinted from [50] under CC-BY license.

The resulting cured materials showed acceptable transparency (with transmittance values beyond 50% in the visible wavelength range). As assessed by flame spread tests carried out in horizontal and vertical configurations, unlike the pristine birch wood—easily burned, leaving a limited residue—the woods treated with the melamine–formaldehyde resin were self-extinguishing (Figure 38). Moreover, as revealed by forced combustion tests performed under 50 kW m^−^^2^ irradiative heat flux, the flame-retardant transparent birch wood showed an increased time to ignition and decreased peak heat release rate and total smoke release (by approximately 14 and 84%, respectively) compared to its non-infiltrated counterpart.

In a further research effort, Chu and co-workers [51] succeeded in obtaining a highly transparent wood (transmittance and haze were as high as 93 and 98%, respectively) by infiltrating it with phosphate ester-poly(ethylene glycol) (synthesized deliberately) in balsa wood delignified by H_2_O_2_ treatment. After curing, the obtained transparent wood showed enhanced thermal stability as assessed by thermogravimetric analyses. Further, in horizontal flame spread tests, the flame-retardant transparent wood achieved self-extinction and a limiting oxygen index as high as 37%. The excellent flame-retardant behavior was also confirmed by forced combustion tests (irradiative heat flux: 50 kW m^−^^2^), which highlighted a decrease in peak heat release rate, heat of combustion, and total heat release by approximately 82, 81, and 84%, respectively, as compared to the same delignified wood infiltrated with an epoxy resin.

### 6.4. Recent Applications in Home Design and Manufacturing 

Very recently, Zhou and Xu exploited the incorporation of a silane coupling agent, namely γ-aminopropyl triethoxysilane, into partially delignified Chinese fir wood, in order to ameliorate the interface between the wood template and the epoxy resin—which the wood was impregnated with—and finally cured at room temperature [52]. The treatment with the silane coupling agent significantly reduced the porosity after the impregnation and curing with the epoxy resin, creating a good interface between the wood and the polymer network. Moreover, the proposed modification accounted for a significant increase in the tensile strength (from approximately 29 to 50 MPa, for pristine and modified wood, respectively) and in the elongation at break (from approximately 1.3 to 3.3%). At the same time, the modified wood exhibited higher light transmittance and lower haze with respect to its original counterpart, hence suggesting its suitability for home design and manufacturing purposes.

## 7. Conclusions and Perspectives

Undoubtedly, wood (nano-)technology is significantly taking advantage of the continuous research work related to transparent wood-based materials. The scientific interest from an academic point of view is nowadays accompanied by increased attention from the industrial world thanks to all the peculiarities of transparent wood and bamboo, which make them the best candidates for the replacement of common glass. Indeed, these materials are able to combine functional and structural performance together, leading to the design of new, advanced systems, suitable for applications in different key industrial sectors.

At present, the scientific knowledge about transparent wood and bamboo has achieved high levels. In fact, thus far, the methods for obtaining them have been remarkably enhanced; the same can be said regarding the functionalization of delignified/modified “woods”, which allows for the design of novel materials suitable for energy-saving and smart glazing, home design, and decorative applications; optoelectronics; sensors; flame-retardant devices, and X-ray shielding, among others.

Apart from all these advantages, the research in transparent woods requires further development to solve some still challenging issues. In particular, at present, it is not possible to set a real value of the refractive index for wood, even after modification, because of its complex structure. As a consequence, the matching between the treated wood and the material to infiltrate sometimes fails or does not achieve the envisaged optical targets. Further research work should deeply investigate the interactions occurring between radiation and wood, aiming at better tuning the optical features of transparent wood based on its structure, morphology, and further modification/functionalization. Establishing robust and reliable structure–property relationships would remarkably widen the application sectors of this up-to-date material.

Furthermore, there are still some limitations concerning the possibility of producing transparent wood with high (or very low) thickness and a large area: these size limitations should be taken into consideration, especially in view of the exploitation of this material at an industrial scale.

Moreover, the availability of resin systems with which to infiltrate wood is still quite restricted; further research work should be focused on this issue, hence widening the overall properties of the transparent wood and subsequently its potential uses.

Finally, some attention should be paid to the development of environmentally friendly strategies for the processing of pristine wood materials, minimizing the utilization of harmful solvents and chemicals, decreasing the reaction times and energy consumption, and reducing the related waste streams.

Looking at the significant enhancements that have taken place in the last few years, it is possible to predict that the above-mentioned issues will find a solution in the very near future, hence increasing the reliability and robustness of transparent wood and paving the way to new application fields. 

## Data Availability

Not applicable.
